# Health Tract, No. 149

**Published:** 1865

**Authors:** 


### HEALTH TRACT, No. 149.

### DIPHTHERIAL DISEASE.

DIPHTHERIA is now a familiar household word; within a very few years, indeed, it had never been heard of by one in a million of the masses. Its fearfully sudden and fatal character, especially among children, makes it of the highest importance that those, at least, who have families should know something of its nature, its causes, its symptoms, and its cure. By examining a great many who have died of it, some general facts have been ascertained, which are of considerable practical interest. Neither chemistry nor the microscope have yet been able to determine that any particular structure of the body is uniformly invaded; nor have any characteristic lesions or destruction of parts been found. One thing, however, is certain: the whole mass of blood is corrupted, is diseased, is destitute of those elements which are necessary to health; it is of a dark, grumous, ugly appearance, filling up every vein and artery, stagnating everywhere, clogging up the whole machinery of life, oppressing the brain, and arresting the flow of nervous energy in every part of the system. No wonder, then, that it crushes out the life, in a very few hours, of feeble childhood, and of older persons who have but little constitutional force.

The three most universally present symptoms of diphtheria in the child are, 1st, general prostration of the whole system; 2d, an instinctive carrying of the hand to the throat; 3d, an offensive breath.

As chemistry has not been able to detect any poisonous ingredient in the atmosphere where diphtheria prevails, we are left to the inference that the air of such a locality is simply deprived of one of its essential health-ingredients; for let it be remembered, that if a little more oxygen were added to the atmosphere we breathe, the very first match that was struck would envelop the world in fire in an instant of time, while if there was a little more nitrogen added to it, all that breathes would suffocate and die within the hour, so easy is it for Omnipotence to wrap the solid globe in flames, or sweep from existence the entire race of animals and man!

Children are almost exclusively attacked with diphtheria because it is a disease of debility—a disease which depresses every power of life—hence the weaker the subject is, the more liable to an attack. An adult has only to maintain himself, the child has to do that and to grow also; hence it has a double call for a constant supply of strength; and a very little deficit in that quality of the air which gives vitality to the blood, is sufficient to make it a fit subject for a diphtheritic attack. The few grown persons who have diphtheria have invariably some scrofulous or other weakening element. Neither a man nor a child in really vigorous health is ever attacked with it; they only suffer who are at the time deficient in stamina — have not the proper resisting power against the inroads of disease.

There is no evidence whatever that diphtheria is "catching." The matter and breath of it have been introduced into the eyes, lips, mouth, arm, etc., of physicians who have generously hazarded these experiments upon themselves, without the slightest ill effects whatever. When several members of a family are attacked, it is not because it is derived one from another, but because of similarity of constitution, habits of life, eating, drinking, air, and other surroundings. It has not as yet been established that a stranger, going into a family where there is diphtheria, takes the disease.

The treatment is a well-ventilated room, sustaining nourishment, and strengthening remedies.

Diphtheria is not innoculable; prevails in every climate, in all seasons, and is equally at home in the princely mansions which line the spacious and well-cleaned street, and in the houses of stenchy courts and contracted alleys. It has no fixed course, may recur any number of times, but only fastens on the scrofulous or those whose constitutions are impaired, or have poor blood; the immediate cause of attack being the breathing of a faulty or defective atmosphere.

